# Supplementary material for: Short-lived calcium carbonate precursors observed in situ via Bullet-dynamic nuclear polarization
Source: Commun Chem. 2024 Sep 17;7:210. doi: 10.1038/s42004-024-01300-5 (PMC11408677; doi:10.1038/s42004-024-01300-5)
Supplement: Supplementary file 2 — Supporting Information [file 42004_2024_1300_MOESM2_ESM.pdf]

Short-lived calcium carbonate precursors observed in situ via Bullet-dynamic nuclear polarization

Ertan Turhan[1,2], Masoud Minaei[3], Pooja Narwal[3], *Benno Meier*[3,4,*], *Karel Kouřil*[3], *Dennis Kurzbach*[1,2,*]

[1]*Institute of Biological Chemistry, Faculty of Chemistry, University of Vienna, Währinger Str. 38, 1090 Vienna, Austria*
[2]*University of Vienna, Vienna Doctoral School in Chemistry (DoSChem), Währinger Str. 42, 1090 Vienna, Austria*
[3]*Institute of Biological Interfaces 4, Karlsruhe Institute of Technology, 76344 Egenstein-Leopoldshafen, Germany*
[4]*Institute of Physical Chemistry, Karlsruhe Institute of Technology, 76131 Karlsruhe, Germany*
**E-Mail: dennis.kurzbach@univie.ac.at; benno.meier@kit.edu*

Supporting Information

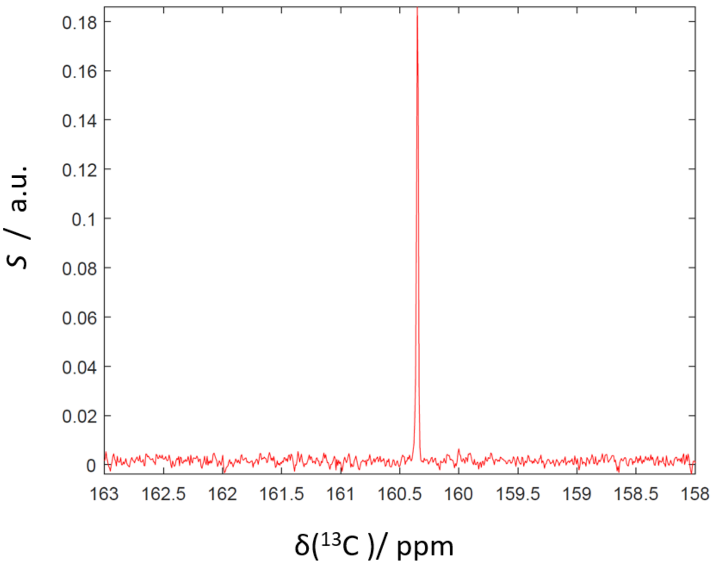

Figure S1. Carbonate reference spectrum in the absence of any calcium ions, *ceteris paribus*.

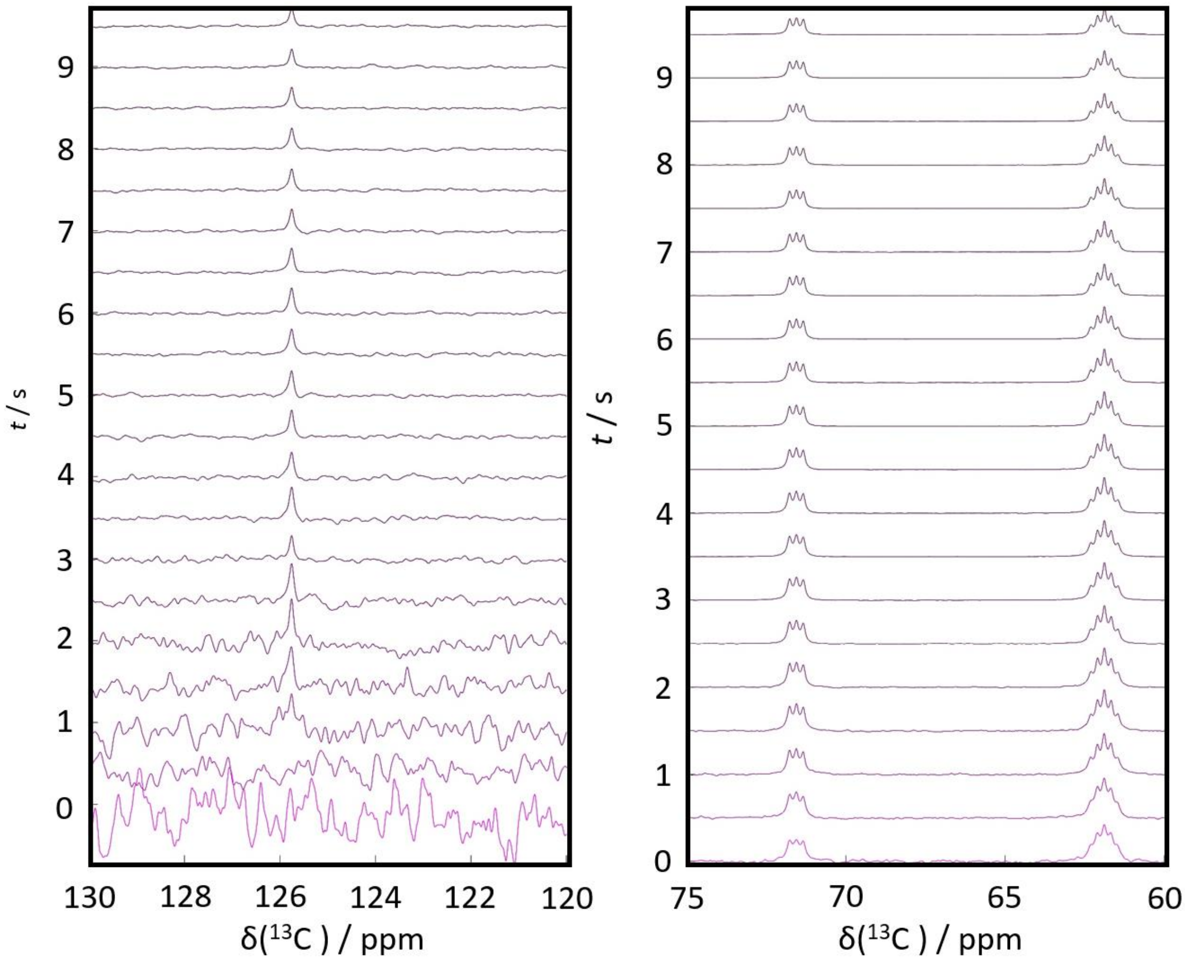

Figure S2. CO_2 spectra (left) and glycerol-d_8 spectra (right) recorded at pH6 after dissolution and mixing for the same experiment reported in the main text. Note that the glycerol signal indicates good shims throughout the entire experiment and that small amounts of CO_2 appeared only ca. 3 s after the start of the detection period as a result of the calcification reaction at pH 6.

Details on the signal correction function.

Supplementary Note 1

Upon injection of the bullet into the liquid sample waiting in the NMR spectrometer, the force of impact causes strong sample convection, resulting in the movement of the target compound in and out of the detected sample volume on the timescale of the NMR detection. Hence, the signal intensity fluctuates even when the sample is homogenous. To correct the resulting biases, we made use of the fact that the water/glycerol solvent mixture entails ^{13}C resonances of the glycerol backbone (Fig. S1). These nuclei are also hyperpolarized, and it can be assumed that glycerol does not take part in the CaC formation as it is not charged and is in large excess compared to carbonate.[1] However, the presence of glycerol may have an impact on the kinetics and dynamics of CaC formation. We determined the time-dependent glycerol signal intensity $S(t)$ by integration of the glycerol signal (Fig. S1). 30 s after dissolution of the bullet, the signal intensities started to decay strictly monoexponentially (Fig. S2). We then fitted the experimental decay for $t > 30$ s to a monoexponential decay function $F(t)$. The resulting function was then back extrapolated to $t = 0$.

To correct the experimentally observed signal intensities for the sample convection, each data point at time *t* after dissolution of the bullet was then multiplied with ratio *F*(*t*) / *S*(*t*).

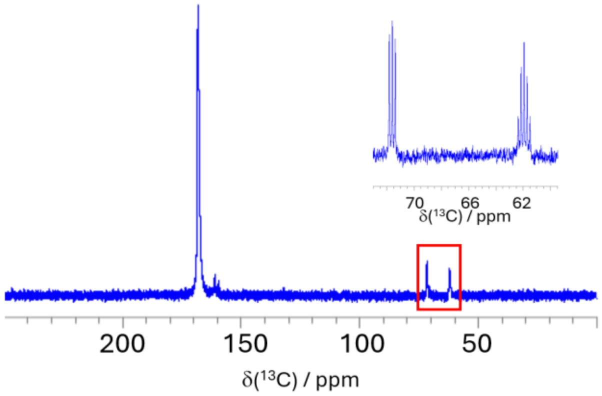

Figure S3. Exemplary ^{13}C spectrum of the entire detected bandwidth. The glycerol signals used to reference the signal intensity are highlighted in red. The inset shows a zoom on the highlighted region. The spectrum was recorded at pH 7, 4 s after mixing of the two solutions.

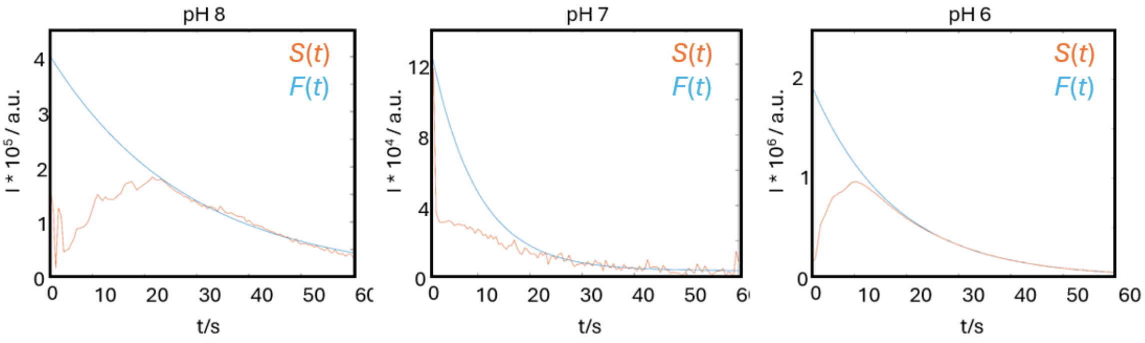

Figure S4. Glycerol signal intensities *S*(*t*) (red) and exponential fit *F*(*t*) to the data at *t* > (blue) for the three different pH values.

Reproductions at varying pH

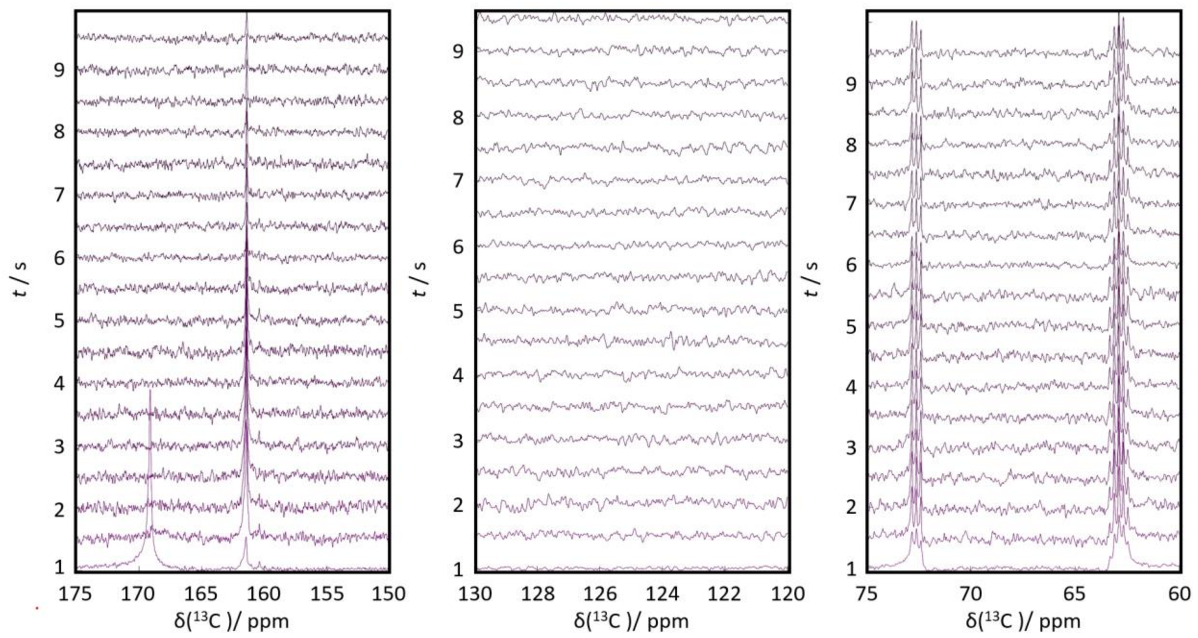

Figure S5. Carbonate (left) spectra and glycerol-d$_8$ spectra (right) recorded at pH 7 after dissolution and mixing. No CO_2 is observed in this experiment (center). Note that the glycerol signal indicates good shims throughout the entire experiment. Most importantly, the three different carbonate species at 159 ppm, 161 ppm, and 168 ppm are reproduced, and hence, no artifact of a specific bullet-DNP experiment or solution conditions.

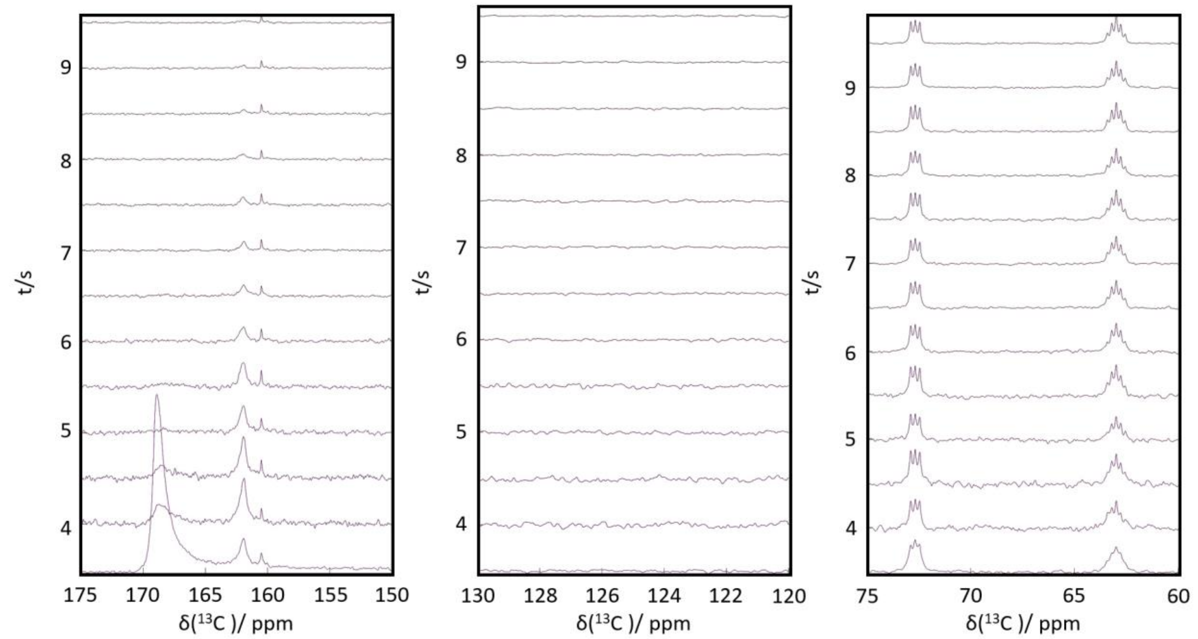

Figure S6. Carbonate (left) spectra, and glycerol-d$_8$ spectra (right) recorded at pH 8 after dissolution and mixing. Again, no CO_2 is observed in this experiment (center), and again, the three observed peaks are reproduced.

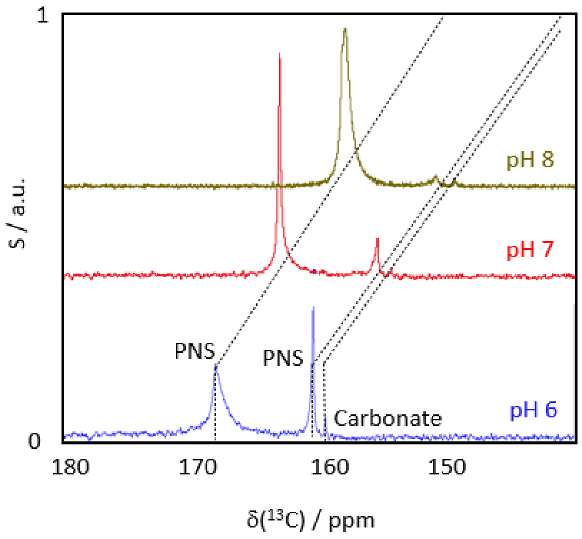

Figure S7. A comparison of the different spectra 2 s after start of detection at pH 6, 7, and 8 led to the signal integral ratios 0.62, 0.87, and 0.96. Note that the line widths of the resonances at 169 ppm are changing with pH. This might be due to varying PNS sizes with pH, as well as changing growth kinetics. Hence, our experiments suggest that not a single PNS size can account for all probed conditions, which is well in line with the reported literature.[1-6]

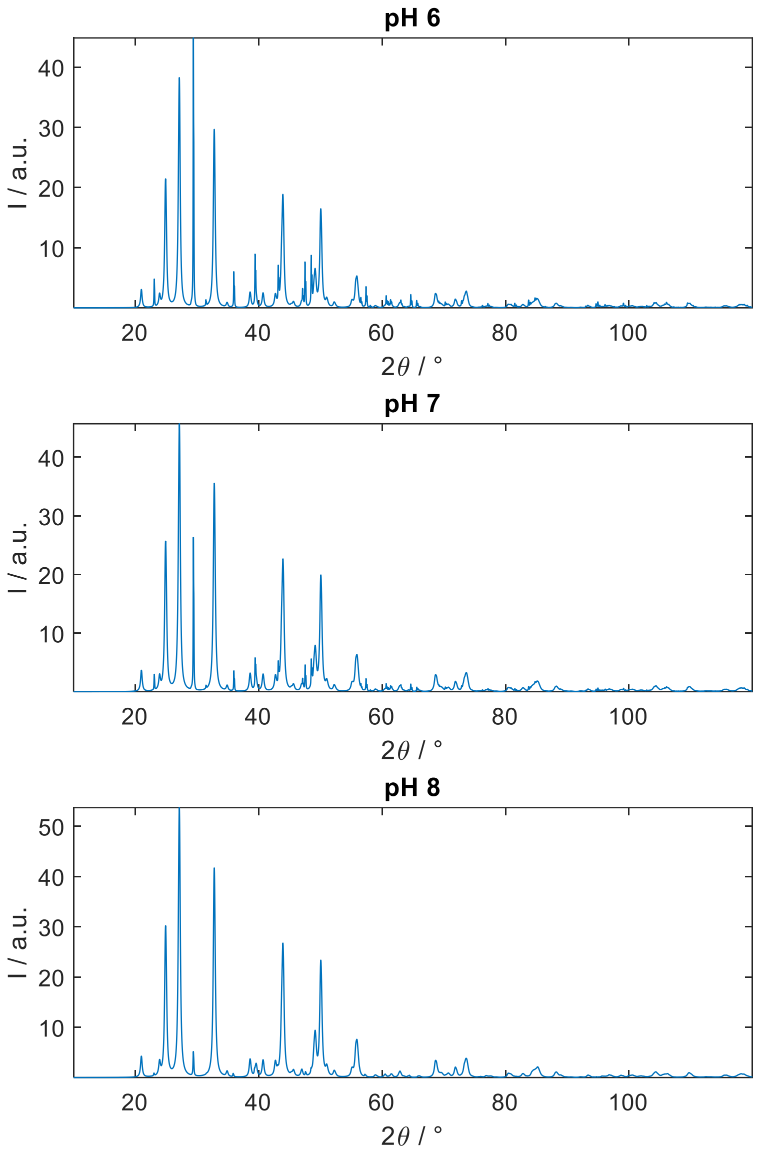

Figure S8. pXRD data of the solids resulting from the Bullet-DNP experiments. A Rietveld refinement yielded for pH 6: 83 % Vaterite (32 nm) and 17 % Calcite (207 nm); for pH 7 91 % Vaterite (30 nm) and 9 % Calcite (205 nm); and for pH 8 97 % Vaterite (31 nm) and 3 % Calcite (95 nm).

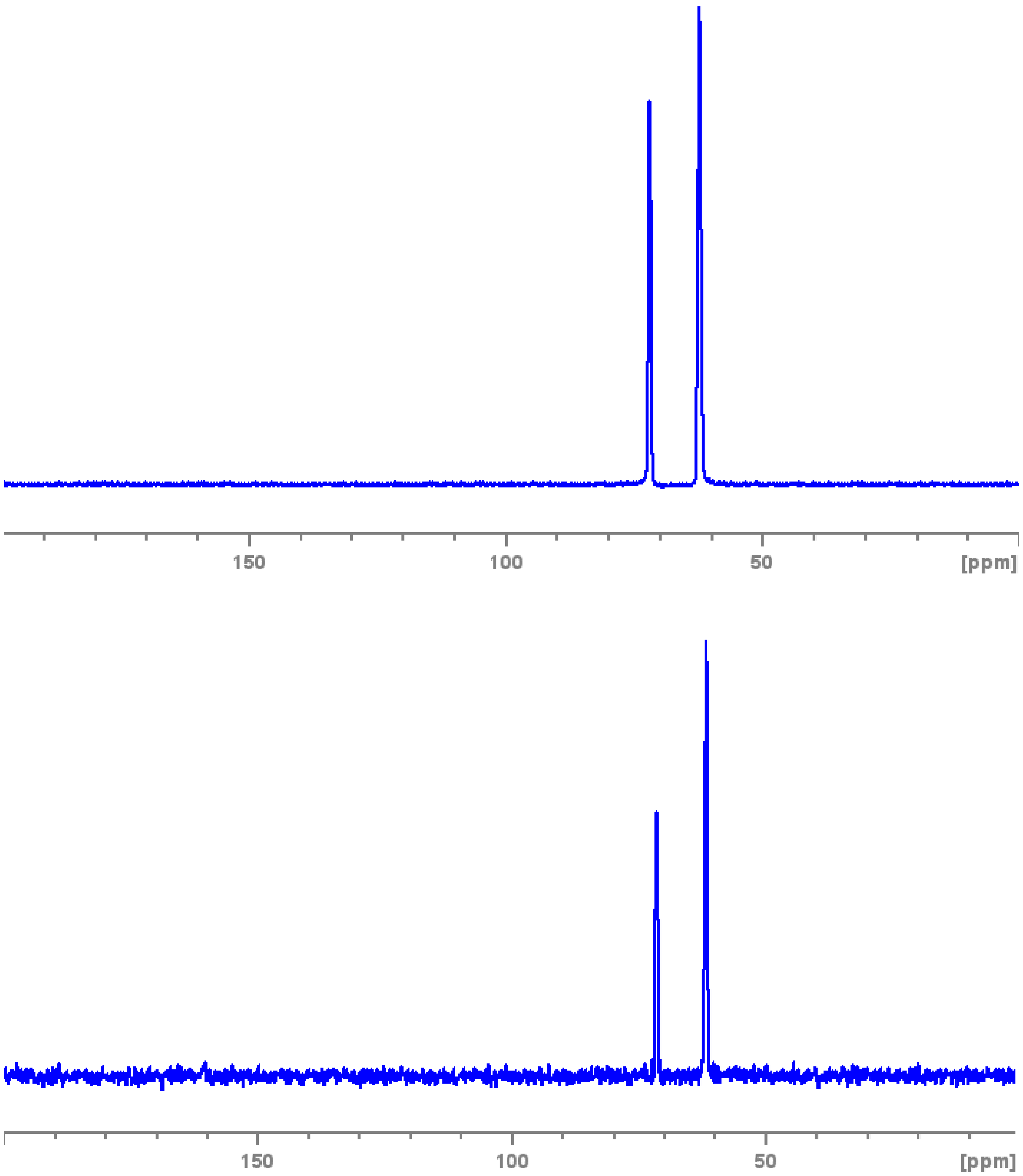

Figure S9. Results for “traditional” DDNP experiments with $KHCO_3$ with the setup at the ENS Paris, France (top) and that at the University of Vienna (bottom). No carbonate species could be observed. Only the signals of glycerol were detected.

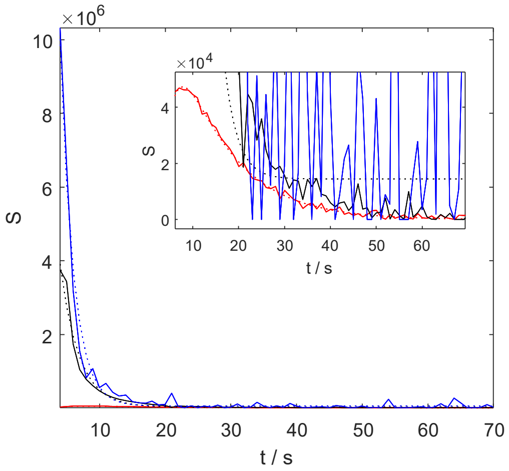

Figure S10. Non-normalized signal intensities corresponding to Fig. 3 of the main text.

Supplementary References

1. Weber, E. M. M.; Kress, T.; Abergel, D.; Sewsurn, S.; Azais, T.; Kurzbach, D., Assessing the Onset of Calcium Phosphate Nucleation by Hyperpolarized Real-Time NMR. *Anal Chem* **2020,** *92* (11), 7666-7673.
2. Henzler, K.; Fetisov, E. O.; Galib, M.; Baer, M. D.; Legg, B. A.; Borca, C.; Xto, J. M.; Pin, S.; Fulton, J. L.; Schenter, G. K.; Govind, N.; Siepmann, J. I.; Mundy, C. J.; Huthwelker, T.; De Yoreo, J. J., Supersaturated calcium carbonate solutions are classical. *Sci Adv* **2018,** *4* (1), eaao6283.
3. Mohammed, A. S. A.; Carino, A.; Testino, A.; Andalibi, M. R.; Cervellino, A., In Situ Liquid SAXS Studies on the Early Stage of Calcium Carbonate Formation. *Part Part Syst Char* **2019,** *36* (6).
4. Huang, Y. C.; Rao, A.; Huang, S. J.; Chang, C. Y.; Drechsler, M.; Knaus, J.; Chan, J. C. C.; Raiteri, P.; Gale, J. D.; Gebauer, D., Uncovering the Role of Bicarbonate in Calcium Carbonate Formation at Near-Neutral pH. *Angewandte Chemie International Edition* **2021,** *60* (30), 16707-16713.
5. Avaro, J.; Moon, E. M.; Schulz, K. G.; Rose, A. L., Calcium Carbonate Prenucleation Cluster Pathway Observed via In Situ Small-Angle X-ray Scattering. *J Phys Chem Lett* **2023,** *14* (19), 4517-4523.
6. Demichelis, R.; Raiteri, P.; Gale, J. D.; Quigley, D.; Gebauer, D., Stable prenucleation mineral clusters are liquid-like ionic polymers. *Nat Commun* **2011,** *2*.
